# Neurological disease progression in adults with ataxia-telangiectasia: a longitudinal study

**DOI:** 10.1093/brain/awag285

**Published:** 2026-08-24

**Authors:** Anke Hensiek, Leonardo Bottolo, May Yung Tiet, Emma Harrison, Elizabeth Ashby, Catarina Olympio, Heather Biggs, Natasha Everett, Katherine Schon, Rita Horvath

**Affiliations:** Department of Clinical Neurosciences, University of Cambridge, Cambridge CB2 0SR, UK; National Clinic for Adults with Ataxia Telangiectasia, Papworth Hospital, Cambridge CB2 0AY, UK; Department of Genomic Medicine, Cambridge Biomedical Campus, University of Cambridge, Cambridge CB2 0QQ, UK; MRC Biostatistics Unit, Cambridge Biomedical Campus, University of Cambridge, Cambridge CB2 0SR, UK; Department of Clinical Neurosciences, University of Cambridge, Cambridge CB2 0SR, UK; National Clinic for Adults with Ataxia Telangiectasia, Papworth Hospital, Cambridge CB2 0AY, UK; Department of Clinical Neurosciences, University of Cambridge, Cambridge CB2 0SR, UK; Department of Clinical Neurosciences, University of Cambridge, Cambridge CB2 0SR, UK; Department of Clinical Neurosciences, University of Cambridge, Cambridge CB2 0SR, UK; Department of Clinical Neurosciences, University of Cambridge, Cambridge CB2 0SR, UK; National Clinic for Adults with Ataxia Telangiectasia, Papworth Hospital, Cambridge CB2 0AY, UK; Department of Clinical Neurosciences, University of Cambridge, Cambridge CB2 0SR, UK; Department of Genomic Medicine, Cambridge Biomedical Campus, University of Cambridge, Cambridge CB2 0QQ, UK; Department of Clinical Neurosciences, University of Cambridge, Cambridge CB2 0SR, UK

**Keywords:** ataxia-telangiectasia, adults, progression, SARA, UK mutation

## Abstract

Ataxia-telangiectasia (AT) is a rare, multisystem neurodegenerative disorder with marked clinical heterogeneity. Longitudinal data on neurological progression remain limited, restricting understanding of disease mechanisms and therapeutic trial design.

We analysed longitudinal Scale for the Assessment and Rating of Ataxia (SARA) data from the largest published adult AT cohort to date (*n* = 82, age > 16 years). Linear mixed-effects models were used to estimate severity at the earliest available neurological evaluation (baseline) and annual progression rates (points/year) in classic and variant AT, and to examine clinical and genetic modifiers.

Neurological impairment progressed with a mean SARA increase of 0.38 points/year. Adults with classic AT were more severely affected than those with variant AT, with SARA scores approximately 8–9 points higher at baseline. However, neurological progression in adulthood was similar between classic and variant AT (0.33 versus 0.41 points/year), which includes 15 individuals with the antisense oligonucleotide targetable c.5763-1050A>G variant.

Although AT subtypes differ substantially in childhood and adolescence, neurological decline converges to a similar pace in adulthood, irrespective of subtype or genotype. SARA remains a useful outcome measure but may underestimate progression in advanced disease and non-cerebellar domains. These findings inform counselling, prognostication and the design of interventional trials, including mutation-specific therapies.

## Teaser

Ataxia-telangiectasia (AT) is a rare, multisystem neurodegenerative disorder with marked clinical heterogeneity. Studying the largest adult AT cohort published to date, Hensiek *et al.* find that although disease severity differs across AT subtypes earlier in life, neurological decline progresses at a similar rate across subtypes in adulthood.

## Introduction

Ataxia-telangiectasia (AT) is one of the most common forms of recessive ataxia worldwide, although it remains rare, with an estimated prevalence of 1 in 40 000–100 000.^1^ The causative gene encodes the ataxia-telangiectasia mutated (ATM) protein, a key regulator of DNA repair. Loss or deficiency of ATM results in immune dysfunction, malignancy predisposition and progressive neurological degeneration, affecting the cerebellum predominantly, but also the basal ganglia and peripheral nerves.^2^

The neurological phenotype of AT is variable. Individuals with the severe classic form, characterized by the complete absence of the ATM protein, typically have a markedly reduced life expectancy and are often wheelchair-dependent by early childhood. By contrast, the milder variant phenotype, associated with residual ATM protein, presents more heterogeneously, with later onset, longer survival and variable severity of neurological involvement.^3,4^

Currently, management of AT is supportive, as no curative treatments exist.^5^ Several novel therapeutic strategies are under investigation, including antisense oligonucleotide (ASO) therapies, which are currently being evaluated for specific ATM variants in ‘*n* of 1’ clinical trials in children.^6^ A deep intronic splice variant c.5763-1050A>G, which is a UK founder mutation,^7^ identified in 15 patients in our cohort, represents a promising ASO target. However, the lack of validated clinical outcome measures and biomarkers poses challenges for therapeutic studies.

In rare diseases such as AT, randomized controlled trials are constrained by small patient populations,^8^ and natural history cohorts provide essential external controls for trials. Moreover, characterizing the trajectory of neurological progression is critical for counselling patients and for understanding the underlying disease mechanisms.^9^

Previous studies of neurological disease progression in AT have primarily focused on children and often been limited by small sample sizes. Data on the rate of neurological change in adults remain scarce.^10^ In this study, we report the largest retrospective longitudinal analysis of adults with AT, assessed using the Scale for the Assessment and Rating of Ataxia (SARA), the most widely applied clinical tool.^11^ Our cohort includes both classic and variant forms of AT, as well as a subgroup of individuals carrying the UK founder mutation.

## Materials and methods

### Study design and patients

#### Probands

Clinical data were retrospectively collected from case notes of 88 adults with AT attending the National Adult Ataxia-Telangiectasia (AT) Service at Papworth Hospital, UK, a nationally commissioned specialist clinic that provides multidisciplinary care for adults (age >16) with AT, where patients from across the UK are reviewed regularly with coordinated clinical assessments, surveillance investigations and ongoing follow-up through the service. Only probands >16 years of age assessed by neurologists from the Papworth Hospital service were included in the analysis (*n* = 82 adults). The baseline assessment was defined as the earliest available neurological evaluation within the National Adult AT Service, where the first SARA score was obtained. All subsequent assessments (follow-up assessments) were included in the longitudinal analysis. Date of symptom onset and date of diagnosis were recorded (Fig. 1A). However, childhood clinical assessments conducted at other centres, and before probands reached the age of 16 when they were first assessed at Papworth Hospital, were not included in the analysis. Disease duration was defined as the interval between age at diagnosis and age at assessment. Past medical history, including previous or current malignancy, was documented in all patients. Level of mobility and age at first wheelchair use were recorded. The neurological examination was performed by neurologists with a special interest in AT. The SARA was recorded.

**Figure 1:**
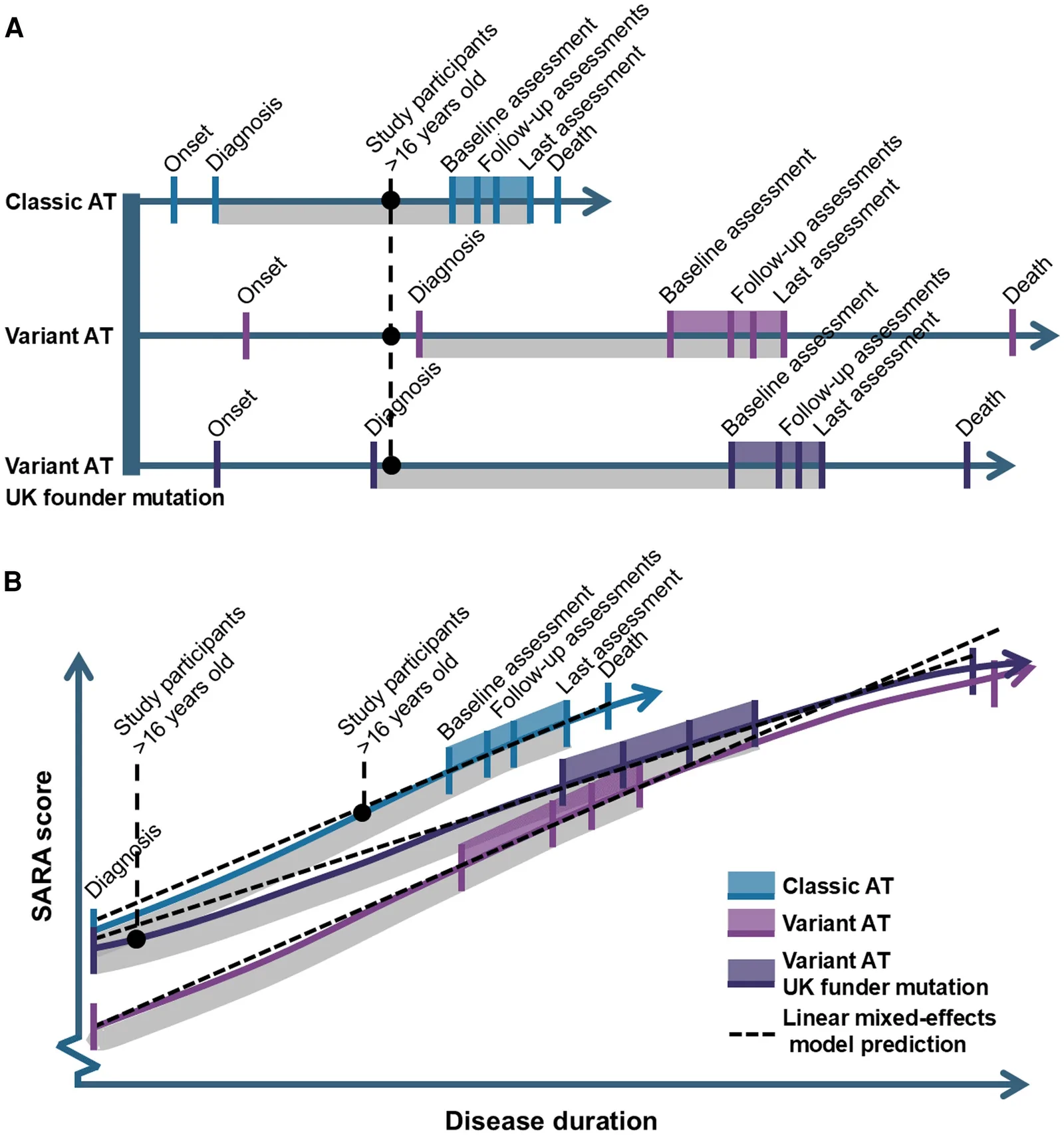
**Study design and disease progression over time.** (**A**) Timeline plots depict, on average (based on cohort characteristics), the age at symptom onset, age at diagnosis, baseline assessment, follow-up assessments and death for individuals with classic AT, variant AT and variant AT carrying the UK founder mutation. Grey bars below the arrows indicate disease duration, defined as the interval between age at diagnosis and age at assessment. Colour-coded bars above the arrows show the time interval between assessments in the different AT groups. Only probands aged >16 years who were assessed by neurologists from the Papworth Hospital service were included in the analysis (dotted black vertical lines). (**B**) Disease progression from the age at diagnosis is shown for individuals with classic AT, variant AT and variant AT carrying the UK founder mutation. On average, patients with classic AT exhibit higher SARA scores at baseline, followed by those with variant AT carrying the UK founder mutation, and then by patients with variant AT. Patients with classic AT tend to have a shorter life expectancy compared with those with variant AT. Grey bars below the arrows indicate disease duration, while colour-coded bars above the arrows show the time interval between assessments in the different AT groups. Linear disease progression (black dotted lines) is similar across groups but slightly faster in variant AT compared with classic AT. AT = ataxia-telangiectasia; SARA = Scale for the Assessment and Rating of Ataxia.

#### Molecular and genetic studies

Patients were classified as having variant AT based on residual ATM protein or retained ATM kinase activity, while classic AT was associated with absent ATM protein. *ATM* mutations were identified by Sanger sequencing or by whole-genome sequencing. A lymphoblastoid cell line was derived from the patients’ blood, and immunoblotting for ATM expression and ATM activity assays were performed as previously described.^12^

#### Ethical considerations

The study was approved by the Health Research Authority (HRA) – 16/HRA/3236/AM01.

### Statistical analysis

SARA score trajectories were evaluated using a frontward timescale,^13^ where year zero corresponds to the age at diagnosis and time represents disease duration. We used the R package *lmer* to estimate the linear trajectories of the SARA score over time. Mixed-effects models^14^ included: (i) quadratic functions of frontward time (time and time squared); (ii) other fixed effects (AT type, presence of ATM protein, history of malignancy and death of the study participant), and their interactions with time and time squared; and (iii) random effects (time and study participant). The models were adjusted for demographic and neurological features (sex, ethnicity and age of diagnosis) and the neurologist who performed the assessment. We also used the R package *lcmm* to estimate latent class mixed-effect models^15^ of the linear trajectories of SARA over time. For details on the statistical models,^16,17^ we refer to the Supplementary material, ‘Methods’ section.

## Results

### Demographic features

The study included 82 patients (43 males/39 females) from 76 families (Table 1 and Supplementary Table 1). The cohort included 43 classic AT and 39 variant AT patients, of which 15 carried the c.5763-1050A>G mutation (UK founder mutation). The mean age at baseline assessment for the whole cohort was 28 years (classic AT: 21 years, variant AT: 35 years). For the first follow-up, the mean time was 16 months (classic AT: 15 months, variant AT: 18 months). The mean number of SARA assessments was four (classical AT: four, variant AT: five). The mean time between SARA assessments was 32 months (classic AT: 28 months, variant AT: 41 months).

**Table 1:** Demographic and neurological features of the cohort, stratified into classic AT, variant AT and variant AT with UK mutation

	All	Classic AT	Variant AT	Variant AT UK founder mutation
Total patients, *n*	82	43	39	15
Total families, *n*	76	–	–	–
Total males, *n*	33	20	13	6
Total females, *n*	49	23	26	9
Age range at onset, years	0–44	0–10	1–44	1–20
Mean (SD) age at onset, years	4 (6)	2 (2)	7 (8)	5 (5)
Age range at diagnosis, years	1–56	1–16	2–56	4–24
Mean (SD) age at diagnosis, years	12 (11)	5 (4)	18 (12)	13 (6)
Age range at baseline assessment, years	16–60	16–52	16–60	16–56
Mean (SD) age at baseline assessment, years	28 (13)	21 (8)	35 (12)	38 (12)
Total patients using wheelchair at baseline assessment, *n*	74	42	32	15
Mean (SD) SARA score at baseline assessment	25 (8)	28 (5)	21 (9)	27 (5)
Mean (SD) number of SARA assessments	4 (3)	4 (2)	5 (3)	4 (3)
Mean (SD) time period between first and second SARA assessments, months	16 (12)	15 (11)	18 (12)	18 (15)
Mean (SD) time period between SARA assessments, months	32 (24)	28 (25)	41 (20)	36 (18)
Disease duration range at the last assessment, years	0–35	0–35	5–33	5–33
Mean (SD) disease duration at the last assessment, years	18 (10)	16 (10)	23 (11)	25 (11)
Total patients with a history of malignancy, *n*	25	13	12	3
Deceased patients, *n*	26	19	7	3
Age range of deceased patients, years	19–64	19–48	48–64	48–62
Mean (SD) age of deceased patients, years	35 (15)	27 (7)	56 (6)	54 (7)

Demographic and neurological features of the 82 patients included in the longitudinal study. Patients are stratified by ataxia-telangiectasia (AT) type (classic, variant and variant with the UK founder mutation). Baseline assessment is defined as the earliest available neurological evaluation within the National Adult AT Service at which a Scale for the Assessment and Rating of Ataxia (SARA) score was obtained. The mean (standard deviation, SD) time in months between SARA assessments is calculated as the mean (SD) of the average time intervals between assessments for each patient. Disease duration is defined as the interval between age at diagnosis and age at assessment.

A total of 25 patients had a history of malignancy, including 13 classic AT and 12 variant AT patients. Nineteen patients with classic AT were recorded as deceased during the retrospective data collection period, while seven patients with variant AT, including three individuals carrying the UK mutation, had died in the same interval (mean age at death: classic AT 27, variant AT 56 and UK founder mutation 53 years).

### Neurological features

The mean SARA score at baseline assessment for the whole patient group was 25, including a mean score of 28 for classic AT, 21 for variant AT and 28 for patients carrying the UK founder mutation. Table 1 summarizes the age of onset, age at diagnosis and disease duration at the last assessment in both classical and variant groups. Supplementary Table 1 provides the information separately for each patient.

### Linear trajectories of the SARA score over disease duration

Linear mixed-effects models (LMMs) with frontward time (where time indicates the disease duration from the age at diagnosis) and its interactions with other fixed effects, time random intercept and patient-specific random slope provided the best fit to the longitudinal data after Box–Cox (power) transformation^4^ (Supplementary Table 2). For the whole cohort, SARA score increases linearly by 0.38 points [*P*-adj < 10^−16^, standard error (SE) = 0.08] every year between diagnosis and assessments. At diagnosis, the LLM predicts that patients with variant AT had an average SARA score roughly 9 points (*P*-adj < 10^−16^, SE = 2.128) lower than those with classic AT (*P*-adj < 10^−16^, α = 23.75, SE = 2.48) (Table 2 and Fig. 2A). At baseline, a difference of 8–9 points between classic and variant AT is predicted. To evaluate the different rates of increase of the SARA score over time between AT types, we estimated their interactions with time. Classical AT patients progress linearly by 0.33 points (*P*-adj = 0.04, SE = 0.16) every year since diagnosis, while variant AT type increases by 0.41 points/year (*P*-adj < 10^−16^, SE = 0.10) (Table 2 and Fig. 2B).

**Figure 2:**
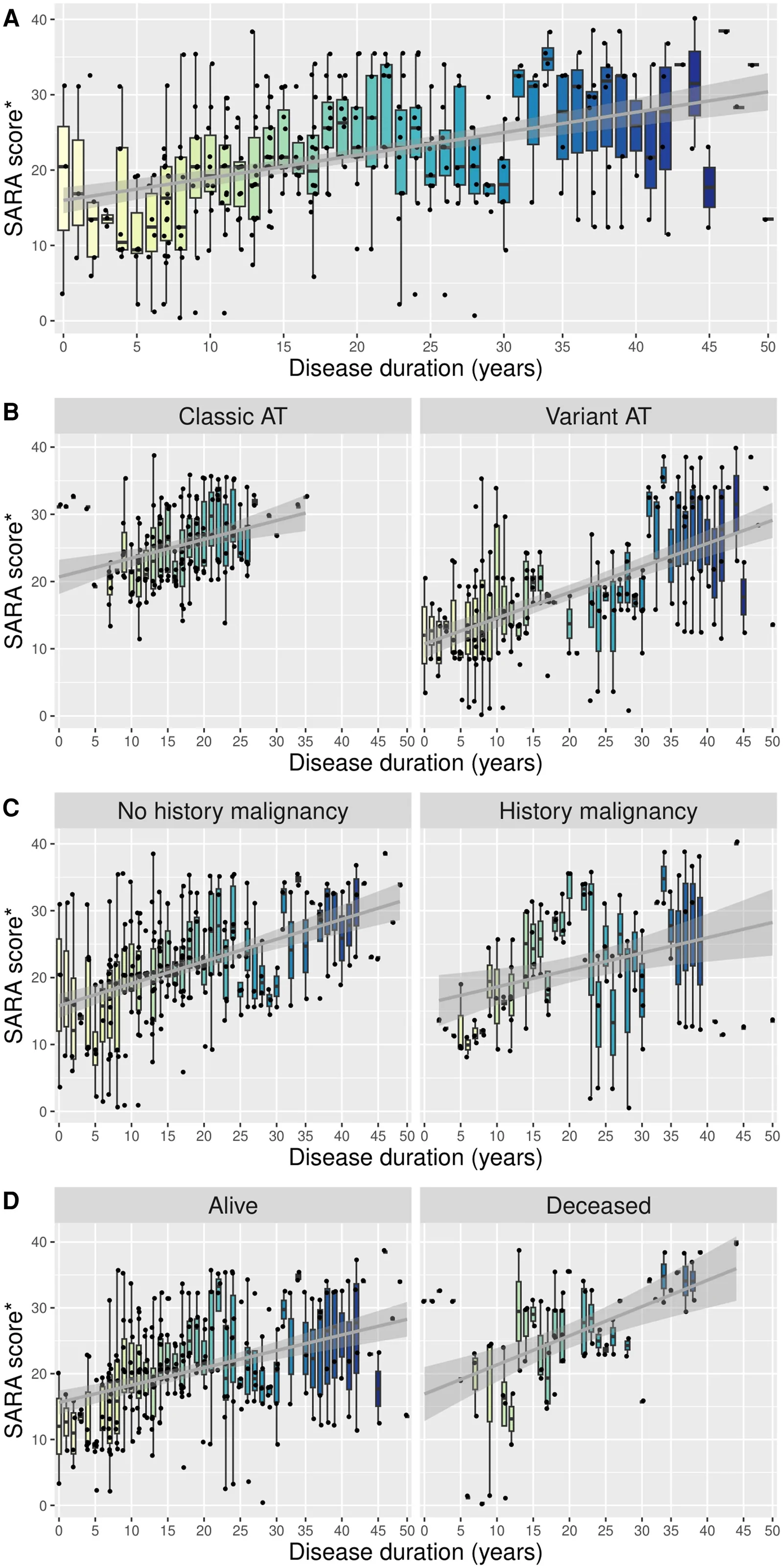
**SARA scores attained by study participants over time.** Black dots represent Box–Cox power-transformed SARA scores (*y*-axis) plotted against disease duration (*x*-axis), defined as the interval (years) between age at diagnosis and age at assessment. Each box plot represents the central tendency (median, horizontal line), dispersion (interquartile range, box length) and skewness of SARA scores for patients assessed within the same disease duration. Box plot colours range from yellow to blue according to disease duration. The solid grey line depicts the linear regression model of disease duration on transformed SARA score, with the 95% confidence interval shown as a grey shaded area. (**A**) Box plots of SARA scores for patients assessed at similar disease duration, stratified by (**B**) AT type (classic AT or variant AT), (**C**) malignancy status (no history of malignancy versus history or presence of malignancy) and (**D**) vital status (alive or deceased). Individual SARA score trajectories at baseline and follow-up assessments are shown in Supplementary Fig. 3. AT = ataxia-telangiectasia; SARA = Scale for the Assessment and Rating of Ataxia.

**Table 2:** Estimated fixed-effects (and interactions) sizes, standard errors and levels of significance for selected linear mixed-effects models

LMM	Fixed effect (and interaction)	Effect size	Standard error	*P-*adj
Frontward time	Intercept	23.754	2.483	<10^−16^
	AT type (variant AT)	−9.037	2.128	<10^−16^
	Time	0.379	0.078	<10^−16^
Frontward time × Sex	Intercept	25.572	3.035	<10^−16^
	AT type (variant AT)	−9.011	2.134	<10^−16^
	Time × Sex (female)	0.435	0.093	<10^−16^
Frontward time × AT type	Intercept	24.567	2.864	<10^−16^
	AT type (variant AT)	−10.436	3.212	0.014
	Time × AT type (classic AT)	0.329	0.117	0.042
	Time × AT type (variant AT)	0.412	0.096	<10^−16^
Frontward time × ATM protein	Intercept	27.902	3.91	<10^−16^
	AT type (variant AT)	−9.15	2.123	<10^−16^
	Time × ATM protein (presence)	0.417	0.08	<10^−16^
Frontward time × Malignancy	Intercept	25.057	2.72	<10^−16^
	AT type (variant AT)	−8.995	2.132	<10^−16^
	Time × Malignancy (no)	0.325	0.092	0.007
	Time × Malignancy (yes)	0.505	0.135	<10^−16^
Frontward time × Vital status	Intercept	24.544	2.564	<10^−16^
	AT type (variant AT)	−8.909	2.129	<10^−16^
	Time × Vital status (alive)	0.33	0.087	<10^−16^
	Time × Vital status (deceased)	0.537	0.148	0.007
Frontward time × AT type × Malignancy	Intercept	26.284	3.077	<10^−16^
	AT type (variant AT)	−10.821	3.195	0.009
	Time × AT type (classic AT) × Malignancy (yes)	0.581	0.166	0.009
	Time × AT type (variant AT) × Malignancy (no)	0.409	0.105	<10^−16^
	Time × AT type (variant AT) × Malignancy (yes)	0.486	0.147	0.018
Frontward time × AT type × Vital status	Intercept	26.894	3.14	<10^−16^
	AT type (variant AT)	−12.315	3.357	<10^−16^
	Time × AT type (classic AT) × Vital status (deceased)	0.506	0.156	0.018
	Time × AT type (variant AT) × Vital status (alive)	0.391	0.097	<10^−16^
	Time × AT type (variant AT) × Vital status (deceased)	0.646	0.205	0.027

Based on Supplementary Table 2, we considered models that included as fixed effects frontward time (i.e. disease duration, defined as the interval between age at diagnosis and age at assessment) or its second-order interactions with sex (Frontward time × Sex), AT type (Frontward time × AT type), ATM protein level (Frontward time × ATM protein), history of malignancy (Frontward time × Malignancy) and vital status (Frontward time × Vital status), as well as third-order interactions with AT type and history of malignancy (Frontward time × AT type × Malignancy) and with AT type and vital status (Frontward time × AT type × Vital status). For significant main effects and interactions, we report the effect size, standard error and adjusted *P*-value (*P*-adj). Full results for significant and non-significant main effects and interactions are presented in Supplementary Table 3. AT = ataxia-telangiectasia; ATM = AT mutated; LMM = linear mixed-effects model.

The trajectories of SARA increase over time are slightly different between patients with history of malignancy (*P*-adj < 10^−16^, *β* = 0.51, SE = 0.14) and those without malignancy (*P*-adj = 0.01, *β* = 0.33, SE = 0.09) (Table 2 and Fig. 2C). It is also marginally different between those who died (*P*-adj = 0.01, *β* = 0.54, SE = 0.15) and those who survived (*P*-adj < 10^−16^, *β* = 0.33, SE = 0.09) (Table 2 and Fig. 2D). We could not confirm statistically significant differences in the increase of SARA score between females and males, as only the female group showed a significant increase (*P*-adj < 10^−16^, *β* = 0.43, SE = 0.01). Similarly, it is not possible to report differences in SARA score progression when the ATM protein is present or absent since only the former was significant (*P*-adj < 10^−16^, *β* = 0.417, SE = 0.08) (Table 2).

SARA score correlates with the disease duration in variant AT patients, both with malignancy (*P*-adj = 0.02, *β* = 0.49, SE = 0.15) or without malignancy (*P*-adj < 10^−16^, *β* = 0.41, SE = 0.11), while in classic AT it is significant only for patients with a history of malignancy (*P*-adj = 0.01, *β* = 0.58, SE = 0.01) (Table 2 and Supplementary Fig. 4A). Similarly, SARA score increases linearly by 0.39 points/year (*P*-adj < 10^−16^, SE = 0.1) in variant AT individuals who survived and by 0.65 points/year (*P*-adj = 0.03, SE = 0.21) amongst deceased patients. In classic AT, it is significant only for patients who had died (*P*-adj = 0.02, *β* = 0.51, SE = 0.15) (Table 2 and Supplementary Fig. 4B).

To check whether the Box–Cox transformation of the longitudinal data may have introduced bias, we repeated the same analysis, treating the SARA assessment as a binomial experiment without any transformation of the recorded SARA scores (Supplementary Table 2). In particular, we confirmed the upward linear trajectory of SARA score in the whole cohort [*P*-adj < 10^−16^, odds ratio (OR) = 1.04, SE = 1.008], although we found that the disease progression measured by SARA was significant only for variant AT (*P*-adj < 10^−16^, OR = 1.05, SE = 1.01) (Supplementary Table 4).

Finally, we investigated the presence of unmeasured (latent) patient subgroups whose combination of demographic, neurological or molecular features could potentially stratify the whole cohort into groups with different SARA trajectories over the disease duration. Using LMMs, we found no evidence of subgroups with a significant effect on SARA score trajectory (Supplementary Table 5).

### Trajectory of SARA score over disease duration for a subset of patients with variant AT carrying the c.5763-1050A>G mutation

We used mixed-effects models to study a subset of study participants with variant AT type who carry the c.5763-1050A>G mutation. This variant alters the splicing of ATM, which can be corrected by ASOs. Specifically, we assessed whether the 15 patients with this mutation had a significantly different rate of increase in SARA score over time compared to 24 variant AT patients who do not carry this variant (Table 1 and Supplementary Table 6). When we restricted the analysis to variant AT patients, we confirmed a significant upward trend of SARA score over time (*P*-adj = 0.03, *β* = 0.28, SE = 0.09) (Supplementary Table 7). Its 95% confidence interval (0.1, 0.46) includes the estimated effect size of frontward time in variant AT patients when the whole cohort is analysed (*P*-adj < 10^−16^, *β* = 0.41, SE = 0.10) (Table 2). For patients without the c.5763-1050A>G mutation (Supplementary Table 1), the SARA score linear trajectory was significant (*P*-adj = 0.01, *β* = 0.40, SE = 0.11). The same results were detected when we correlated disease duration and the presence of the c.5763-1050A>G mutation by using generalized LMMs (Supplementary Tables 5 and 8).

## Discussion

Here we describe the natural history of neurological decline, as measured by the SARA score, in the largest published cohort of adults with AT. We demonstrate a gradual deterioration in neurological function across all adults with AT, both classic and variant forms, with the SARA score worsening by an average of 0.38 points/year. As expected, and consistent with the known disease progression, adults with classic AT were significantly younger and had more severe disease, with higher SARA scores, compared to those with the milder variant form. At the earliest available neurological evaluation, after adjusting for demographic and neurological features of the study participants, and the neurologist who performed the assessment, patients with variant AT had a predicted SARA score roughly 8–9 points lower than those with classic AT. However, when the rate of progression was examined, the annual deterioration was quite similar in both groups: 0.33 points/year in classic AT and 0.41 points/year in variant AT.

These findings suggest that once adulthood is reached, the rate of neurological progression is not greatly different between classic and variant AT. While classic AT typically presents in early childhood with rapid progression in the first years of life and most children becoming wheelchair-dependent by primary school age, our data indicate that the rate of progression slows afterwards. Although adults continue to deteriorate, the annual rate of decline, as measured by the SARA score, is comparable between classic and variant AT, and even marginally higher in AT variant type. History of malignancy and vital status of the patient provided only a mild indication that the disease progression is higher in these individuals, and in particular, those with variant AT. Furthermore, we could not confirm significant differences in progression rates between individuals carrying the UK founder mutation and other adults with variant AT. These findings have important implications for patient counselling, prognostication and the design and sample size calculations of therapeutic studies in adults with AT, where the SARA score is commonly employed as an outcome measure. Based on the results of our analysis and the characteristics of the cohort (on average, four SARA assessments for each study participant over three years), the power to detect a slope of 0.38 with 82 subjects (and a type I error of 0.05) is 0.79. To detect a 50% reduction in disease progression in a 3-year trial with 80% power and the same type I error, the required sample size would be approximately four times larger (≈337). In a 1-year trial, the required sample size to achieve 80% power with the same type I error would be approximately forty times larger (≈3360). However, if the trial is extended to 5 years, the required sample size to detect a 50% reduction in disease progression would be only 97 subjects. Thus, statistical power is influenced by both the number of subjects and the number of SARA assessments, and it is likely that international multicentre studies would be required for adequately powered trials.

A recent paper reported that serum AFP levels increased over time in individuals with classic AT until the age of approximately 18 years, when a plateau was reached.^18^ We measured AFP levels at the time of diagnosis, which were increased as expected. However, AFP levels were not measured longitudinally in a systematic way in our cohort as AFP was not considered previously as a useful biomarker of disease severity.^19^ In light of these new data, it is recommended to monitor AFP levels longitudinally.

The progression rate of the SARA score in our adult AT cohort was slower than reported in spinocerebellar ataxias (SCA1, SCA2, SCA3).^20^ This apparent paradox may arise from the multisystemic nature of AT and from the adult study population. It is possible that the progression rate would be higher in childhood AT. A recent study on Friedreich’s ataxia recorded that the SARA score, together with other clinical scores [Friedreich’s Ataxia-Activities of Daily Living Rating Scale (FARS-ADL) and Inventory of Non-Ataxia Signs (INAS)] worsened significantly over time. Repeat length and baseline SARA score emerged as the strongest predictors of progression. CSF neurofilament light chain was elevated in younger patients and declined with age but did not correlate with severity or progression. These results support the premise that the baseline SARA score is a robust predictor of progression.^21^

Our results should be interpreted in the context of certain limitations. The neurological phenotype of AT is complex, particularly in the variant form, with involvement of multiple systems including the peripheral nervous system, extrapyramidal features and cognitive decline.^22,23^ In this study, we used the SARA score to quantify neurological disability. The SARA is a standardized and validated tool for assessing ataxia severity, widely used both for clinical monitoring and as an outcome measure in clinical trials.^24,25^ The score is designed to capture key motor functions relevant to cerebellar dysfunction, such as gait, coordination, balance and speech. However, it is not sensitive to other neurological abnormalities that are also relevant in AT, such as neuropathic weakness, cognitive changes, oculomotor abnormalities or extrapyramidal symptoms.^26,27^ While we selected the SARA score for its ease of administration and established role in natural history studies and clinical trials, our analysis does not capture progression in non-cerebellar systems, where additional deterioration may occur.

Our study demonstrates a linear deterioration of ataxic symptoms over time, which appears similar in adults with classic and variant AT. However, these findings are limited by the fact that the SARA scale is not strictly linear.^28^ Because the scale places significant emphasis on gait and balance, changes in neurological function may be more difficult to detect once patients become non-ambulant. This ceiling effect was particularly relevant for patients with classic AT, who were already non-ambulant at their first assessment in adulthood, thereby entering the study with high baseline SARA scores.

Finally, we chose to model the disease progression linearly to provide an easy-to-interpret rate of increase over time, although non-linear models may have fit the longitudinal data better and provided different rates according to the time elapsed from the diagnosis.

Our cohort also included a subgroup of patients carrying the c.5763-1050A>G UK founder mutation. We could not establish significant differences in disease progression compared with the broader variant AT group, although statistical power was limited by the small sample size. Notably, an ASO therapy targeting other ATM variants is currently in clinical trials in children. To better understand disease progression and identify reliable outcome measures for adults with this mutation, a prospective natural history and biomarker study is ongoing in Cambridge, UK.

In summary, our study demonstrates that adults with AT experience a measurable slow decline in neurological function, with similar progression rates in classic and variant forms. The SARA score remains a useful outcome measure for adult AT, although it may underestimate changes in non-cerebellar domains and in advanced disease. These findings have direct implications for counselling, trial design and the development of future therapies, including mutation-specific approaches such as ASOs.

## Supplementary Material

awag285_Supplementary_Data

## Data Availability

The raw data supporting the findings of this study are available from the corresponding author upon reasonable request, subject to institutional and ethical approval requirements.
