# Supplementary material for: Neurological disease progression in adults with ataxia-telangiectasia: a longitudinal study
Source: Brain. 2026 Aug 24;149(10):3387–94. doi: 10.1093/brain/awag285 (PMC13634639; doi:10.1093/brain/awag285)
Supplement: Supplementary file 1 [file awag285_supplementary_data.pdf]

Supplementary Material

Neurological disease progression in adults with ataxia telangiectasia - a longitudinal study

Anke Hensiek[1,2,*] Leonardo Bottolo,[3,4,*], May Tiet[1,2], Emma Harrison[1], Elizabeth Ashby[1], Catarina Olympio[1], Heather Biggs[1], Natasha Everett[2], Katherine Schon[1,3] and Rita Horvath[1,2]

Author affiliations:

1 Department of Clinical Neurosciences, University of Cambridge, Cambridge, CB2 0SR, UK

2 National Clinic for Adults with Ataxia Telangiectasia, Papworth Hospital, Papworth Road, Cambridge, CB2 0AY, UK

3. Department of Genomic Medicine, Cambridge Biomedical Campus, University of Cambridge, Cambridge CB2 0QQ, UK

4 MRC Biostatistics Unit, Cambridge Biomedical Campus, University of Cambridge, Cambridge, CB2 0SR, UK

[*]These authors contributed equally

Methods

SARA score trajectories were assessed by using a frontward timescale, in which year zero corresponds to the age at diagnosis, and time reflects disease duration defined as the interval between age at diagnosis and age at assessment.

We employed mixed-effects models[1] to estimate the linear trajectory of the SARA score over time. These models included:

i. quadratic functions of frontward time (time and time squared),
ii. other fixed effects (AT type, presence of ATM protein, history of malignancy and vital status) and their interactions with time and time squared,
iii. random effects (time and study participant),
iv. and were adjusted for demographic (sex and ethnicity) and neurological (age of diagnosis) features of the patients and other assessment covariates (examiner).

Given the assumption of normality of the error term in linear mixed-effects models (LMM), we transformed the SARA score by using Box-Cox power transformation[2], implemented in the R software, version 4.5, package *MASS*, version 7.3-65. In generalised linear mixed-effects models (GLMM), we treated SARA assessment as a binomial experiment where the cumulative score ranges from zero (no ataxia) to 40 (most severe ataxia). We also assumed that the scores in each of the eight items of the SARA assessment are independent.

We checked in LLM and GLMM whether cross or nested random effects provide a better fit to the longitudinal data. In cross-random effects, we assumed that changes in SARA score between different years of follow-up are independent of the variation of SARA score within each patient, i.e., we considered time and study participant random intercepts. In nested random effects, we assumed that changes in SARA score between different years of follow-up may differ across patients, i.e., we considered a time random intercept and patient-specific random slope. We also evaluated the effect of frontward time and its quadratic function (time and time squared), their second-order interactions with sex, AT type, presence of ATM protein, history of malignancy and vital status and their third-order interaction with AT type and history of malignancy, and AT type and vital status. We used Akaike Information Criterion (AIC) and Bayesian Information Criterion (BIC)[3] to select the best LLM and GLMM model between crossed and nested random effects, between frontward time and its quadratic function and whether higher-order interactions provide a better fit of the longitudinal data.

To fit mixed-effects models, we employed the R package *lmer*, version 1.1-37, and specified a binomial family with a default logistic link function in GLMM.

In all models, the significance of the fixed effects and interactions was declared at 5% Bonferroni-adjusted *P*-values.

Only for LMM, we visually inspected the diagnostic plots, in particular the normal quantile-quantile plot, to check if greater than expected levels of standardised residuals were present. Observations with large standardised residuals were removed, and the mixed-effect models were refitted before estimating AIC and BIC and selecting significant fixed effects and interactions. They were also removed in the other subsequent analyses (GLM and LCMM).

We used the R package *lcmm*, version 2.2.1, to detect latent class mixed-effect models[4] of the trajectories of SARA score over time. We specified nested random effects, and only frontward time was included in the regression model while all other fixed effects (and second-order interactions with frontward time) and covariates were identical to LMM and GLMM. For each model, we checked whether one latent component on the SARA score trajectory (which coincides with LMM) or two latent classes fit the longitudinal data better. We restarted the algorithm 10 times with different seeds to avoid the results depending on the initialisations and reported the best model out of 10 random (seed) starts. For the Box-Cox power transformation of the SARA score, we used the function hlme. For the original SARA score, we employed the function lcmm, which is designed to model the link function non-parametrically by a spline curve, selecting the default value of five equidistant nodes for the spline.

Finally, we used mixed-effects models (LLM and GLM) for a subset of patients with variant AT who carry the splice variant c.5763-1050A>G (UK funder mutation). Since patients with variant AT have detected levels of ATM protein, mixed-effects models included:

i. frontward time,
ii. other fixed effects (UK founder mutation, history of malignancy and vital status) and their interactions with time,
iii. random effects (time and study participant),
iv. and were adjusted for demographic (sex and ethnicity) and neurological (age of diagnosis) features of the patients and other assessment covariates (examiner).

Only for LMM, we visually inspected the diagnostic plots, in particular the normal quantile-quantile plot, to check if greater than expected levels of standardised residuals were present. Observations with large, standardised residuals were removed, and the mixed-effect models

were refitted before estimating AIC and BIC and selecting significant fixed effects and interactions. They were also removed in the subsequent GLMM analysis.

References

1. Pinheiro José C. & Douglas M. Bates. *Mixed-Effects Models in S and S-PLUS*. *Mixed-Effects Models in S and S-PLUS* (Springer, New York, 2000).

2. Box, G. E. P. & Cox, D. R. An analysis of transformations. *Journal of the Royal Statistical Society, Series B* 26, 211–252 (1964).

3. Claeskens, G. & Hjort, N. L. *Model Selection and Model Averaging*. *Model Selection and Model Averaging* (Cambridge University Press, 2008).

4. Proust-Lima, C., Philipps, V. & Liquet, B. Estimation of extended mixed models using latent classes and latent processes: The R package lcmm. *J Stat Softw* 78, 1–56 (2017).

Figures

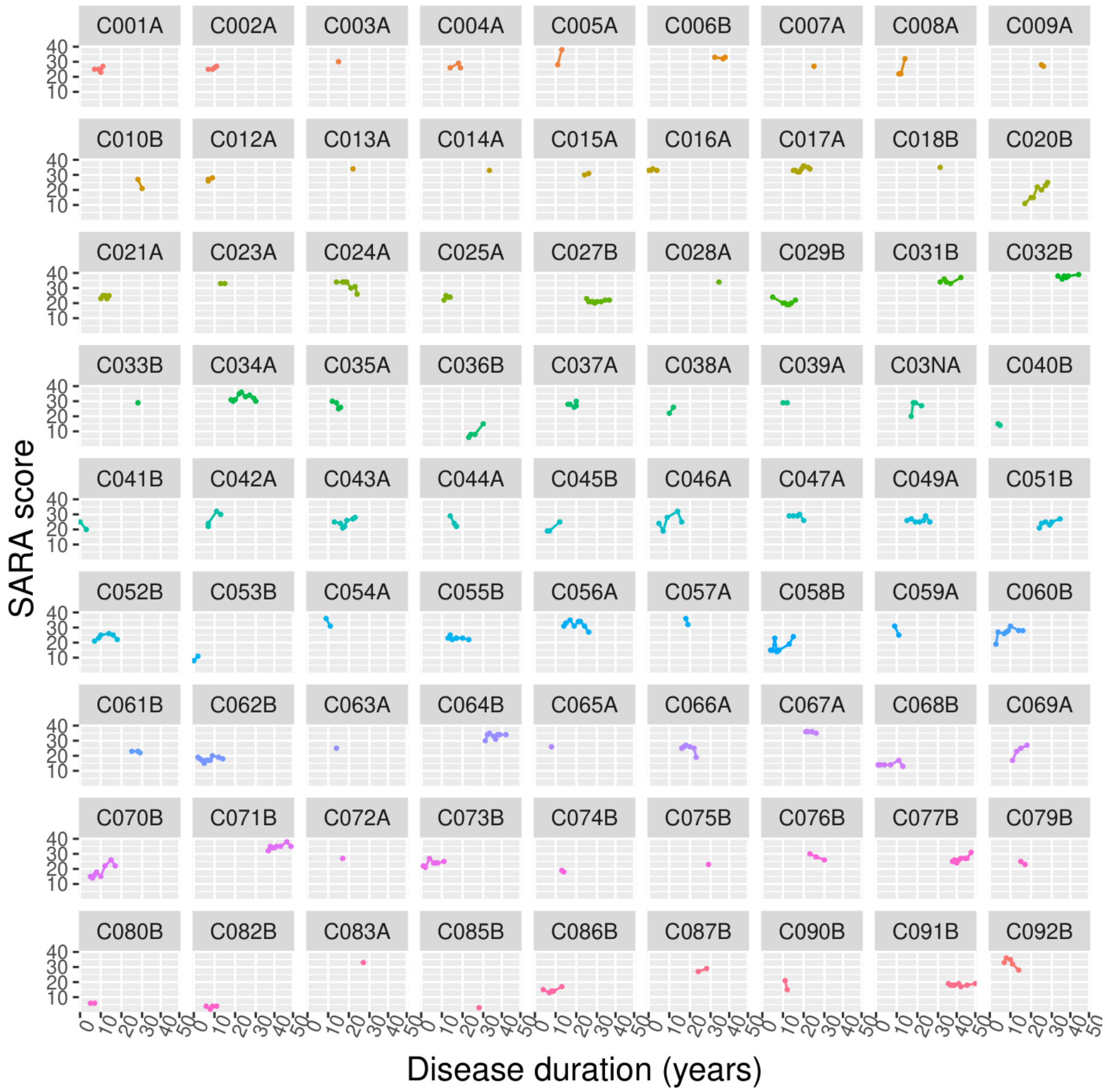

Figure S1. SARA score trajectories for each study participant over time. Each panel represents the SARA score (*y*-axis) attained by an individual patient across the disease duration (*x*-axis), defined as the interval (in years) between age at diagnosis and age at assessment. Panels are ordered from left to right and top to bottom according to the study participant ID shown at the top of each panel.

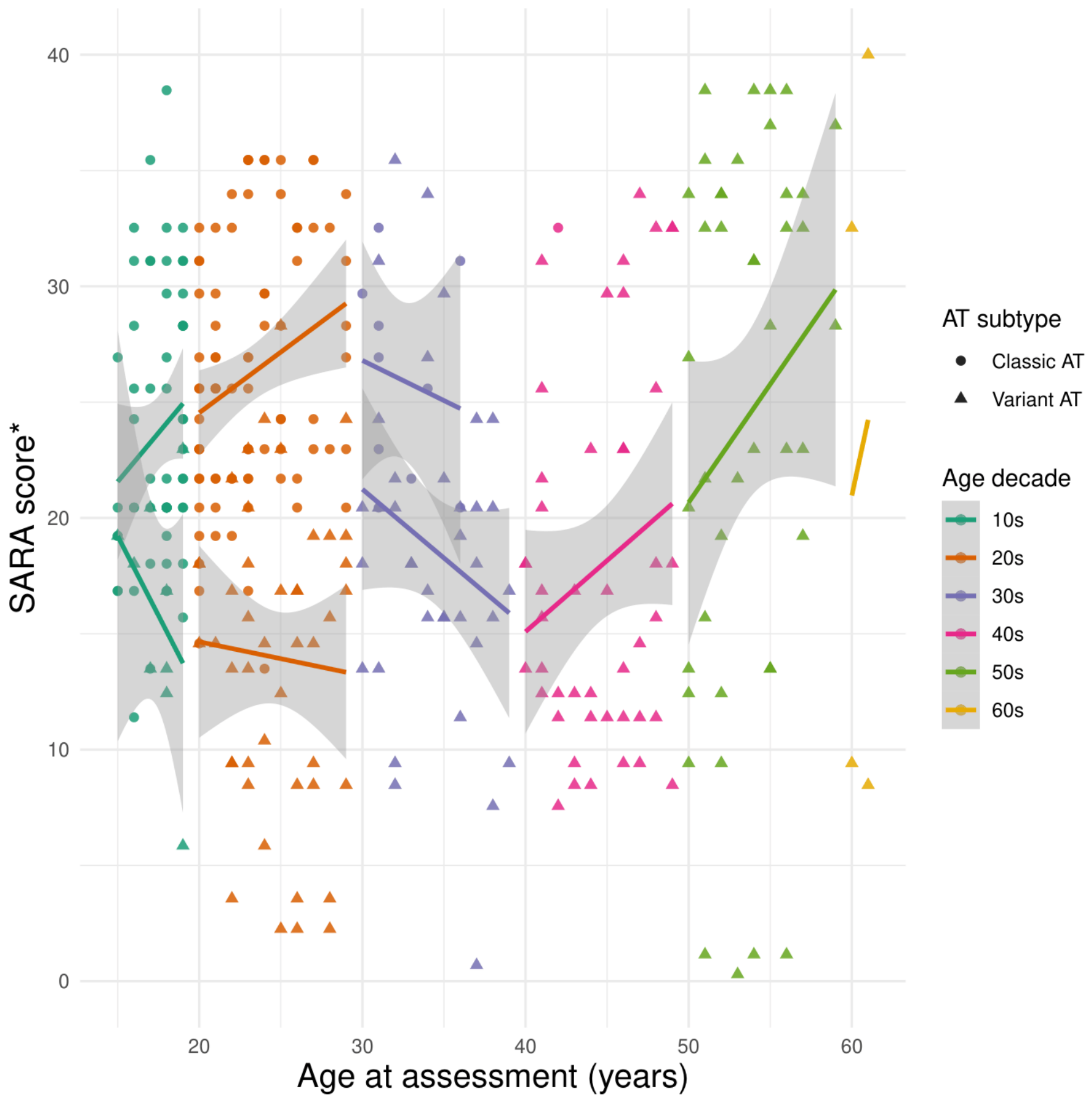

Figure S2. Scatterplot of SARA scores against age at assessment (without adjustment for demographic and neurological features and the neurologist who performed the assessment), with points coloured by age decade and shaped to distinguish classic from variant AT. Box–Cox power–transformed SARA score (y-axis), indicated with "*".

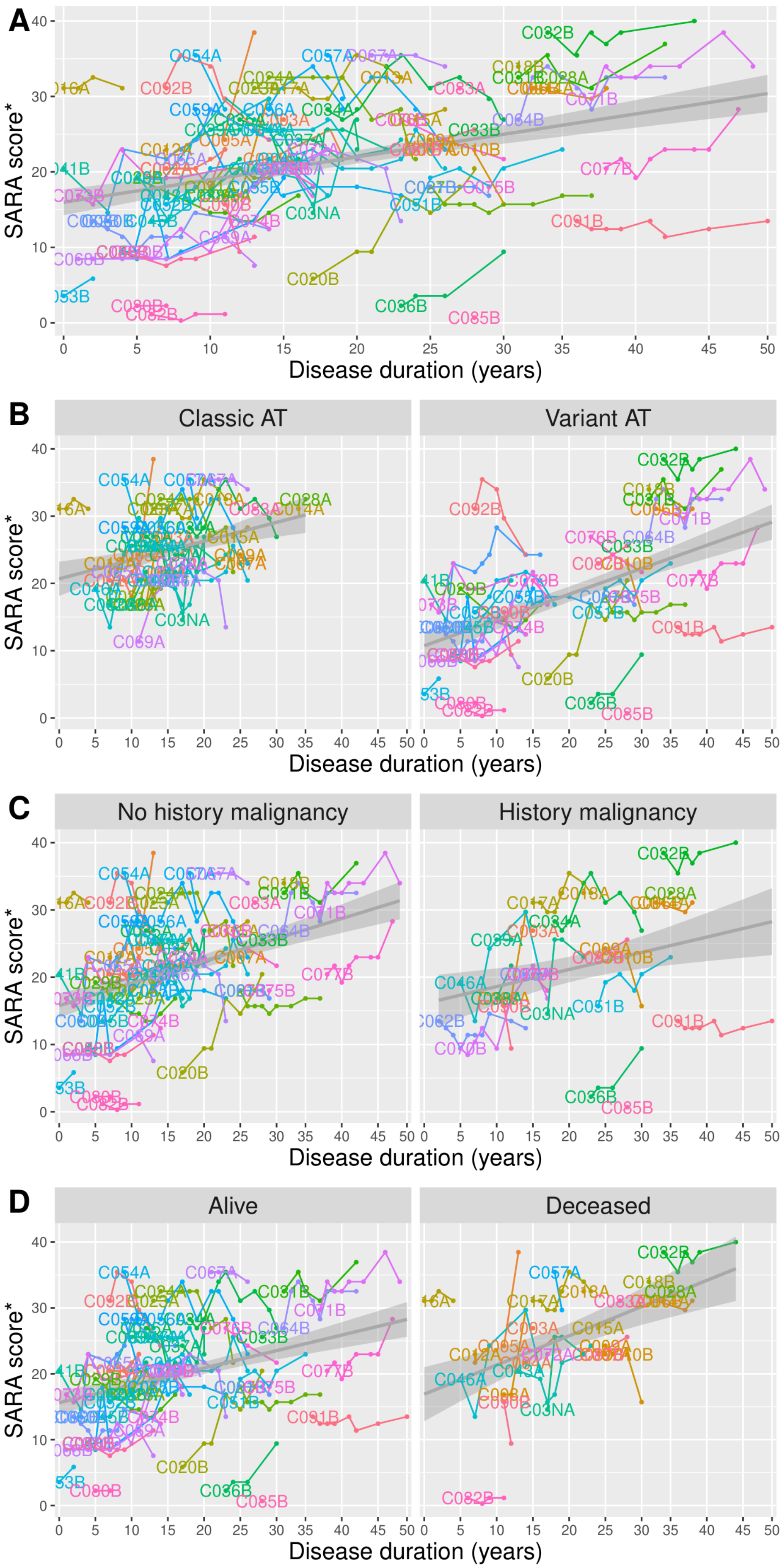
A
B
C
D
SARA score*
Disease duration (years)
Classic AT
Variant AT
No history malignancy
History malignancy
Alive
Deceased

Figure S3. SARA score trajectory for each study participant over time. Colour-coded dots and lines indicate the Box–Cox power–transformed SARA score (*y*-axis, indicated with "*") attained by an individual patient across the disease duration (*x*-axis), defined as the interval (in years) between age at diagnosis and age at assessment. The same colour code is used for the study participant ID. The straight grey line depicts the linear regression model of disease duration on transformed SARA score, with the 95% confidence interval shown as a grey shaded area. (**A**) SARA score for each study participant over time, stratified by (**B**) AT type (classic AT or variant AT), (**C**) malignancy status (no history of malignancy vs. history or presence of malignancy), and (**D**) vital status (alive or deceased).

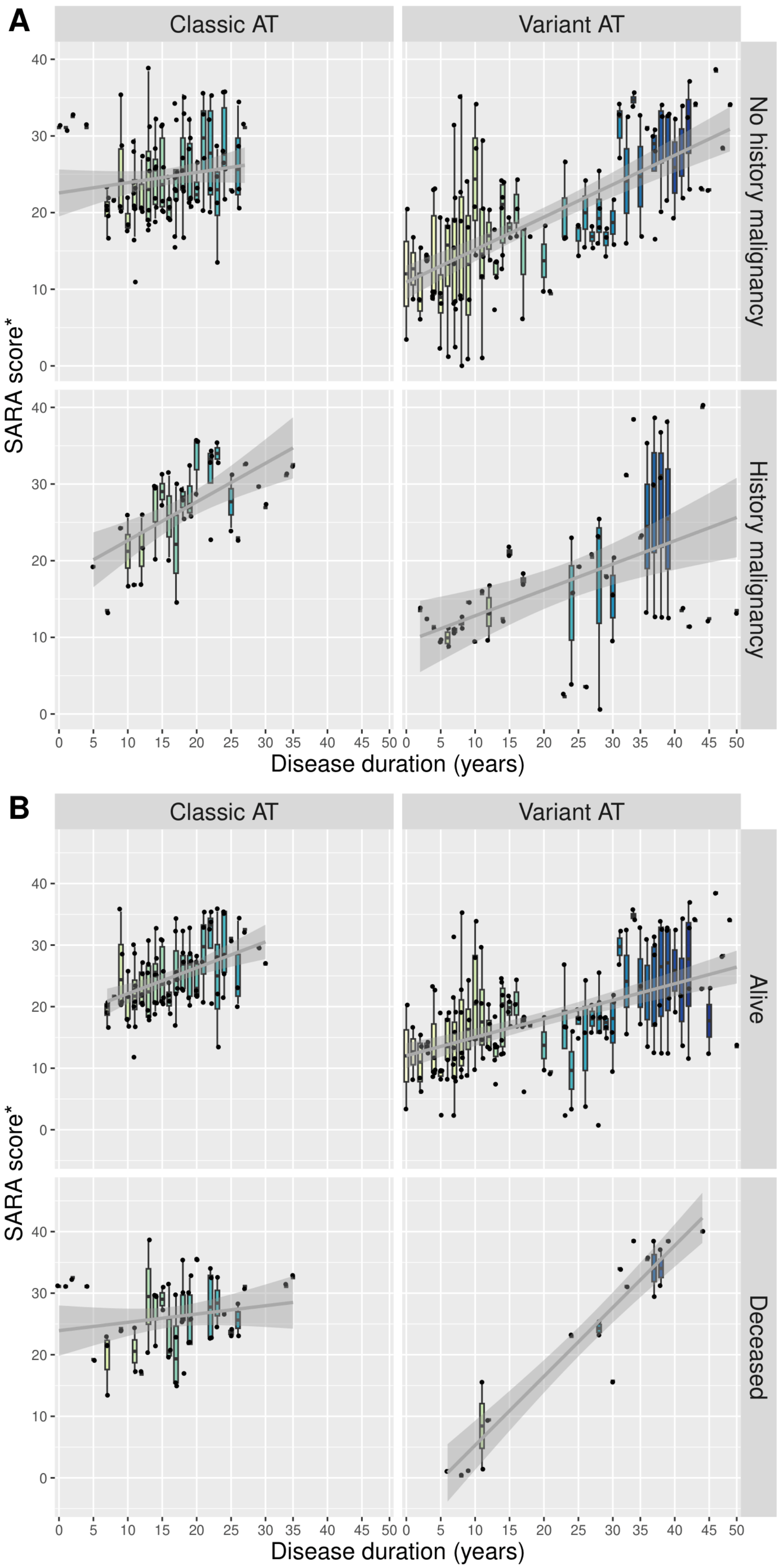
A
Classic AT
Variant AT
No history malignancy
History malignancy
SARA score*
Disease duration (years)
B
Classic AT
Variant AT
Alive
Deceased
SARA score*
Disease duration (years)

Figure S4. SARA scores stratified by AT type, malignancy status, and vital status of study participant over time. Black dots represent Box–Cox power–transformed SARA scores (*y*-axis) plotted against disease duration (*x*-axis), defined as the interval (in years) between age at diagnosis and age at assessment. Each boxplot summarises the central tendency (median, horizontal line), dispersion (interquartile range, box length), and skewness of SARA scores for patients assessed within the same disease duration. Boxplot colours range from yellow to blue according to disease duration. The solid grey line depicts the linear regression model of disease duration on transformed SARA score, with the 95% confidence interval shown as a grey shaded area. (**A**) Boxplots of SARA scores for patients assessed at similar disease duration and stratified by AT type (classic AT or variant AT) and malignancy status (no history of malignancy vs. history or presence of malignancy). (**B**) Boxplots of SARA scores for patients assessed at similar disease duration and stratified by AT type (classic AT or variant AT) and vital status (alive or deceased). Individual SARA score trajectories over time, stratified by AT type, malignancy status, and vital status, are shown in **Figure S5**.

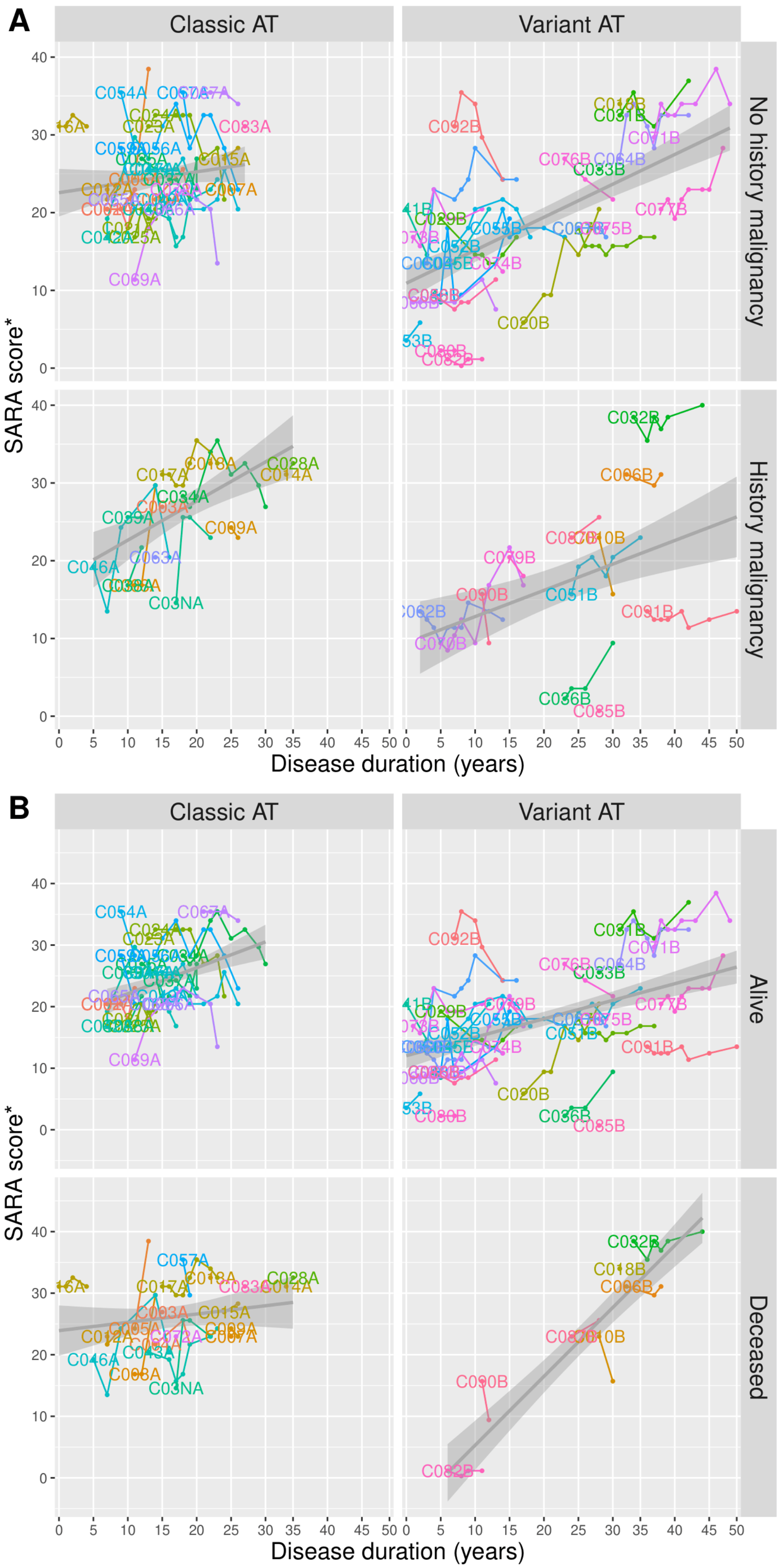

A
Classic AT
Variant AT
No history malignancy
History malignancy
SARA score*
Disease duration (years)
B
Classic AT
Variant AT
Alive
Deceased
SARA score*
Disease duration (years)

Figure S5. SARA score trajectories for each study participant stratified by AT type, malignancy status, and vital status over time. Colour-coded dots and lines indicate the Box–Cox power–transformed SARA score (*y*-axis, indicated with "*") attained by an individual patient across the disease duration (*x*-axis), defined as the interval (in years) between age at diagnosis and age at assessment. The same colour code is used for the study participant ID. The straight grey line depicts the linear regression model of disease duration on transformed SARA score, with the 95% confidence interval shown as a grey shaded area. SARA score for each study participant over time, stratified by (**A**) AT type (classic AT or variant AT) and malignancy status (no history of malignancy vs. history or presence of malignancy), and (**B**) AT type (classic AT or variant AT) and vital status (alive or deceased).

Tables

Supplementary Table 1. Individual patient data.

Study ID	ATM mutations	Age at onset of neurological disease	Age at loss of independent walking	Age at wheelchair dependence	Age at last assessment	ATM protein level	AFP level	Presence or history of malignancy
C001A	c.5631_5635delCTCGCinsA;p.Phe1877Leu fs*39 & c.4397G>C;p.Arg1466Pro	4	10	Not recorded	20	Residual protein present	542	No malignancy
C002A	c.5631_5635delCTCGCinsA;p.Phe1877Leu fs*39 & c.4397G>C;p.Arg1466Pro	4	10	Not recorded	20	Residual protein present	607	No malignancy
C003A	c.6916delAG & c.4850T>C;Leu>proline	0.5	Not recorded	8	18	Absent	Not recorded	Stomach cancer
C004A	c.6997_6998insA;p.Thr2333fs & c.5712_5713ins;p.Ser1905fs	Not recorded	Not recorded	8	24	Absent	Not recorded	Not recorded
C005A	c.3802delG	0.75	Not recorded	8.5	18	Absent	Not recorded	
C006B	c.5763-1050A>G;p.Pro1922fs & c.9022C>T;p.Arg3008Cys	2	16	Not recorded	43	Residual protein present	Not recorded	Oesophageal cancer
C007A	c.790delT;p.Tyr264Ilefs*12 & c.2483delA;p.Lys828Serfs*8	1	11	15	30	Absent	Not recorded	No malignancy
C008A	c.1966A>C & c.1968-71delT	4	Not recorded	9	18	Absent	482	T-cell acute lymphoblastic leukemia (T-ALL)
C009A	c.5228C>T;p.Thr1743lle & c.1782delA;p.Val596fs	1	5	Not recorded	29	Residual protein present	403	Oesophageal cancer

C010B	c.5763-1050A>G;p.Pro1922fs & c.8786+1G>A;p.Gly2891fs	2	Not recorded	Not recorded	48	Residual protein present	210	Pancreatic cancer
C012A	c.7913G>A;p.Trp2638*exon55 & c.5848G>C;p.A1a1950 pro	8	Not recorded	Not recorded	26	Residual protein present	491	No malignancy
C013A	c.2639-?_2838+?del;p.Gly880fs & c.8206_8207delAA;p.Asn2736fs	1	Not recorded	9	32	Absent	Not recorded	T-cell acute lymphoblastic leukemia (T-ALL) & Breast cancer
C014A	c.7638_7646del9;p.Arg2547_Ser2549del & c.497del166	1	Not recorded	Not recorded	36	Residual protein present	85	Thyroid cancer
C015A	c.5363delG & c.2251-2A>T;p.Ser751_Lys792del	1	Not recorded	Not recorded	31	Absent	Not recorded	No malignancy
C016A	c.2284_2285delCT;p.Leu762fs & c.8786+1G>A;p.Gly2891fs	Not recorded	Not recorded	Not recorded	20	Absent	Not recorded	No malignancy
C017A	c.3349C>T;p.Gln1117* & c.5493_5494dupTG;p.Glu1832Valfs*3	1	9	Not recorded	24	Absent	Not recorded	Leukaemia
C018B	c.5763-1050A>G;p.Pro1922fs & c. 2284_228delCT;p.Leu 762fs	5	Not recorded	Not recorded	56	Residual protein present	Not recorded	No malignancy
C020B	c.1563_1564delAG;p.Glu522llefs*21 & c.8480T>G;p.Phe2827Cys	2	Not recorded	Not recorded	33	Residual protein present	430	No malignancy
C021A	c.8395-8404del10;p.Phe2799LysfsX4 & c.2550G>A;p.Lys750 (exon16)	1	11	Not recorded	28	Residual protein present	319	No malignancy
C023A	c.3102T>G;p.Tyr1034*	1	Not recorded	Not recorded	19	Absent	569	No malignancy
C024A	c.3576G>A;p.Ser1135_Lys1192del58 & c.2413C>T;p.Arg805Ter	1	10	Not recorded	26	Absent	333	Not recorded
C025A	c.217_218delGA;p.Glu73fs & c.67C>T;p.Arg23Ter	3	10	Not recorded	21	Residual protein present	581	Not recorded

C027B	c.5763-1050A>G;p.Pro1922fs & Duplication exon 55-63	5	16	Not applicable	45	Residual protein present	179	No malignancy
C028A	c.T2C;p.Met1Thr & c.640_640delT;p.Ser214ProfsTer16	2	10	Not recorded	43	Residual protein present	291	Breast cancer
C029B	c.5763-1050A>G;p.Pro1922fs & c.1563_1564delAG;p.Glu522Ilefs*21	1	11	Not recorded	26	Residual protein present	69	No malignancy
C030A	c.5515C>T;p.Gln1839Ter & c.2251-1G>C;p.Ser751GlufsTer20	0	8	Not recorded	25	Absent	390	T-cell prolymphocytic leukemia (T-PLL)
C031B	c.3403del174	11	17	Not recorded	59	Residual protein present	61	No malignancy
C032B	c.3403del174	11	14	Not recorded	61	Residual protein present	40	Bladder cancer
C033B	c.5763-1050A>G;p.Pro1922fs & c.9022C>T;p.Arg3008Cys	2	8	Not recorded	41	Residual protein present	156	No malignancy
C034A	c.4389_4389delT;p.Phe1463fs & c.7928 del83	0	Not recorded	Not recorded	30	Absent	259	Breast cancer
C035A	c.5763-1G>C & c.216_217delAG;p.Glu73fs	3	Not recorded	11	18	Absent	226	No malignancy
C036B	c.590G>A;p.Gly197Gln	16	Not recorded	Not recorded	32	Residual protein present	511	Breast cancer
C037A	c.5290delC;p.Leu1764fs & c.1564_1565delGA;p.Glu522IlefsTer43	3	Not recorded	Not recorded	24	Absent	410	No malignancy
C038A	c.6116A>T;p.Glu2039Val & c.6300C>G;p.Tyr2100Ter	10	Not recorded	Not recorded	20	Residual protein present	134	T cell lymphoma
C039A	c.6116A>T;p.Glu2039Val & c.6300C>G;p.Tyr2100Ter	4	Not recorded	Not recorded	23	Residual protein present	182	T cell lymphoma

C040B	c.3284_3285ins201;p.Leu1096TrpfsTer62 & c.7638_7646del9;p.Arg2547_Ser2549	12	Not applicable	Not applicable	60	Residual protein present	10	No malignancy
C041B	c.5228C>T;p.Thr1743Ile & c.5763-1050A>G;p.Pro1922fs	8	12	16	26	Residual protein present	81	No malignancy
C042A	c.6047A>G;p.Asp2016Gly & c.3576 G>A;p.Ser1135_Lys1192del58	8	Not recorded	17	28	Residual protein present	104	No malignancy
C043A	c.5290_5290delC;pLeu1764fs & c.2250 G>A;p.Glu709_Lys750del42	2	10	Not recorded	35	Residual protein present	374	No malignancy
C044A	c.1442delT;p.Leu481Ter & c.9022C>T;p.Arg3008Cys	2	11	Not recorded	23	Residual protein present	283	No malignancy
C045B	c.1564_1565delGA;p.Glu522Ilefs*43 & c.8418+681A>G;p.Glu2807Valfs*4	3	13	Not recorded	36	Residual protein present	50	No malignancy
C046A	c.1442delT;p.Leu481Ter 7 & c.9022C>T;p.Arg3008Cys	2	Not recorded	Not recorded	27	Residual protein present	314	Thyroid cancer
C047A	c.7013T>C;p.Leu 2338Pro & c.6056A>G;p.Tyr2019Cys	1	Not recorded	7	23	Residual protein present	165	No malignancy
C049A	c.8098A>T;p.Lys2700X & c.8506A>G;p.Met2836Val	0	13	Not recorded	36	Residual protein present	356	No malignancy
C050A	c.1290_1291delTG;p.Cys430Ter & c.5177+5G>A;p.Glu1669ValfsTer12	7.5	Not applicable	Not applicable	52	Absent	100	No malignancy
C051B	c.5763-1050a>g;p.Pro1922fs & c.6136delC;p.Leu2046fs*1	7	13	Not recorded	57	Residual protein present	145	Breast Sarcoma
C052B	IVS18+6T>A	16	20	Not recorded	39	Residual protein present	<4	No malignancy

C053B	c.7271T>G;p.Val2424Gly & c.7638_7646delTAGAATTTC;p.Arg2547_Ser2549del	17	Not recorded	Not applicable	24	Residual protein present	5	No malignancy
C054A	c.6198+538_6347+131del1002	1	Not recorded	Not recorded	19	Residual protein present	184	No malignancy
C055B	c.8494C>T;p.Arg2832Cys & c.1844T>C;p.Leu615Pro	3	13	Not recorded	27	Residual protein present	715	No malignancy
C056A	c.7665delinsGTGA;p.His2555GInfsX2 & c.2T>C;p.Met1Thr	0	8	Not recorded	32	Residual protein present	296	No malignancy
C057A	c.3102T>G;p.Tyr1034Ter	1	Not recorded	Not recorded	25	Absent	428	No malignancy
C058B	c.6490G>T;p.Glu2164X & c.7184A>T;p.Asp2395Val	1	43	Not recorded	58	Residual protein present	26	Family history of Oesophageal cancer. None for self.
C059A	c.3856delT;p.Cys1286Alafs*7	6	7	Not recorded	19	Absent	546	No malignancy
C060B	c.2T>C; p.Met1Thr & c.9139 C>T;p.Arg3047*	2	11	Not recorded	31	Residual protein present	118	No malignancy
C061B	c.5763-1050A>G;p.Pro1922fs & c.6412_6414del AG	8	12	Not applicable	44	Residual protein present	162	No malignancy
C062B	c.9023G>A;p.Arg3008>His & c.1066-6T>G	1	40	Not recorded	53	Residual protein present	43	Prostate cancer
C063A	c.823delT;pLeu275fsTer1 & c.3754_3755TA[3];p.Lys1253llefsTer4	1	10	Not recorded	18	Absent	530	Ovarian cancer
C064B	c.2T>C;p.Met1Thr & c.5763-1050A>G;p.Pro1922fs	5	30	Not recorded	60	Residual protein present	49	No malignancy
C065A	c.169T>G;p.Trp57Gly	1	2	Not recorded	21	Residual protein present	199	No malignancy

C066A	c.5623C>T;p.Arg1875X & c.2250ins3;p.Lys750_Ser75ins3	1	5	Not recorded	26	Absent	266	No malignancy
C067A	c.2483delA;p.Lys828SerfsTer8 & c.3802delG;p.Val1268Ter	0	Not recorded	9	29	Absent	366	No malignancy
C068B	c.5644C<T;p.Arg1882	14	Not applicable	Not applicable	39	Residual protein present	8	No malignancy
C069A	c.7638_7646del9;p.Arg2547-Ser2549del & c.9022C>T;p.Arg3008Cys	3	Not recorded	Not recorded	29	Residual protein present	101	No malignancy
C070B	c.103C.T;p.Arg35Ter & c.7271T>G;p.Val2424Gly	5	22	Not recorded	34	Residual protein present	20	Vulval cancer
C071B	c.5763-1050A>G;p.Pro1922Leufs*9 & c.8418+2_5delTGAG;p.Val2757_Met2806del	9	11	Not recorded	57	Residual protein present	332	No malignancy
C072A	c.7638_7646del9;p.Arg2547_Ser2549del & c.5825C>T;p.Ala1942	1	5	12	20	Residual protein present	663	No malignancy
C073B	c.3G>T;p.Met1Ile & c.7271T>G;p.Val2424Gly	4	21	Not recorded	32	Residual protein present	22	No malignancy
C074B	c.5763-1050A>G;p.Pro1922fs & c.8491del7	3	16	16	21	Residual protein present	90	No malignancy
C075B	c.5763-1050A>G;p.Pro1922fs & c.5763-1050A>G;p.Pro1922fs	20	32	Not recorded	49	Residual protein present	76	No malignancy
C076B	c.5763-1050A>G;p.Pro1922fs & c.6199-6_6227del35;p.Ala2067Glnfs*10	3	16	Not recorded	41	Residual protein present	146	No malignancy
C077B	c.5763-1050A>G;p.Pro1922fs & c.9139C>T;p.Arg3047*	1	17	Not recorded	59	Residual protein present	164	No malignancy
C079B	c.1158delG;p.Lys387fs & c.6807G>A	2	18	Not recorded	39	Residual protein present	146	Breast cancer

C080B	c.delA;p.Asp130fs*23 & Skipping of exons 33 and 34;p.Asn1638llefs*9	6	Not applicable	Not applicable	28	Residual protein present	Not recorded	No malignancy
C082B	c.1A>G;p.Met1Val & c.8672G>A;pGly2891Asp	44	Not recorded	Not applicable	62	Residual protein present	10	Breast cancer
C083A	c.480del5	1	10	Not recorded	29	Absent	Not recorded	No malignancy
C085B	c.2730_2731insAG;p.Thr910fsTer & c.5005_5006Ins255;p.Gly1669 GlyfsTer2	1	Not applicable	Not applicable	19	Residual protein present	3	T-cell acute lymphoblastic leukemia (T-ALL)
C086B	c.6490G>T;p.Glu2164* & c.7184A>T;p.Asp2395Val	8	37	Not recorded	48	Residual protein present	74	No malignancy
C087B	p.7271T>G;p.Val2424Gly & c.8266A>T;p.Lys2756*	16	25	Not recorded	48	Residual protein present	22	Pancreatic cancer
C090B	c.5623C>T;p.Arg1875* & c.743G>T;p.Arg248Leu	2	46	Not recorded	52	Residual protein present	299	Liver cancer
C091B	c.6490G>T;p.Glu2164X & c.7184A>T;p.Asp2395Val	5	39	Not recorded	52	Residual protein present	66	Chronic myeloid leukaemia (CML)
C092B	c.875C>T;p.Pro292Leu & c.5129_1060del9263;p.Glu1669Aspfs*16	2	22	Not recorded	38	Residual protein present	220	No malignancy

Clinical and molecular characteristics of individuals with Ataxia-Telangiectasia included in the study. For each participant, the table reports Study ID, ATM genotype, age at onset of neurological disease, age at loss of independent walking, age at wheelchair dependence, age at last clinical assessment, ATM protein level, AFP level, and presence or history of malignancy. ATM = Ataxia Telangiectasia Mutated; AFP = Alpha-fetoprotein. “Not recorded” indicates data not available; “Not applicable” indicates the event did not occur.

Supplementary Table 2. Estimated Akaike Information Criterion and Bayesian Information Criterion for each mixed-effects model considered

		LMM				GLMM			
		Cross random effects		Nested random effects		Cross random effects		Nested random effects	
		**Frontward time**	**Quadratic function frontward time**	**Frontward time**	**Quadratic function frontward time**	**Frontward time**	**Quadratic function frontward time**	**Frontward time**	**Quadratic function frontward time**
Frontward time	AIC	1828.9	1841.241	**1808.599**	1819.660	1731.176	1733.174	**1721.36**	1723.32
	BIC	1881.917	1898.045	**1865.402**	1880.251	1780.406	1786.19	**1774.376**	1780.124
Frontward time × Sex	AIC	1830.814	1853.61	**1811.423**	1828.957	1731.39	1734.677	**1722.603**	1726.553
	BIC	1887.617	1917.987	**1872.014**	1897.121	1784.407	1795.267	**1779.407**	1790.93
Frontward time × AT type	AIC	1831.082	1847.099	**1812.311**	1824.856	1730.497	1731.573	**1722.151**	1723.665
	BIC	1887.885	1911.476	**1872.902**	1893.02	1783.514	1792.163	**1778.954**	1788.042
Frontward time × ATM protein	AIC	1826.42	1846.101	**1810.109**	1827.486	1728.091	1731.656	**1721.046**	1724.466
	BIC	1883.224	1910.478	**1870.699**	1895.651	1781.107	1792.246	**1777.85**	1788.843
Frontward time × Malignancy	AIC	1832.311	1851.51	**1811.156**	1831.299	1732.125	1733.872	**1722.44**	1726.27
	BIC	1889.114	1915.887	**1871.746**	1899.464	1785.142	1794.462	**1779.243**	1790.648
Frontward time × Vital status	AIC	1829.988	1852.46	**1810.802**	1830.186	1728.924	1731.116	**1720.568**	1724.363
	BIC	1886.791	1916.837	**1871.393**	1898.35	1781.941	1791.706	**1777.372**	1788.74
Frontward time ×AT type × Malignancy	AIC	1833.841	1867.467	**1817.054**	1844.928	1727.665	1730.842	**1722.165**	1726.218
	BIC	1898.218	1946.991	**1885.218**	1928.24	1788.255	1806.58	**1786.542**	1805.742
Frontward time × AT type × Vital status	AIC	1830.688	1853.175	**1813.936**	1834.25	1729.637	1732.018	**1722.834**	1726.119
	BIC	1891.278	1925.126	**1878.313**	1909.988	1786.441	1800.183	**1783.425**	1798.07

In linear mixed-effects models (LMMs) and generalized linear mixed-effects models (GLMMs), we evaluated both crossed and nested random-effects structures. For each random-effects specification, we assessed whether frontward time or its quadratic term (time and time squared) provided a better fit to the longitudinal data. For every model (defined by the choice of mixed-effects framework (LMM or GLMM), random-effects structure (crossed or nested), and functional form of frontward time (linear or quadratic)), we evaluated model fit using the Akaike Information Criterion (AIC) and Bayesian Information Criterion (BIC). Rows in the table report the estimated AIC and BIC values for models including frontward time (or its quadratic term) and its second-order interactions with sex (Frontward time × Sex), AT type (Frontward time × AT type), ATM protein level (Frontward time × ATM protein), history of malignancy (Frontward time × Malignancy), and vital status of the study participants (Frontward time × Vital status), as well as third-order interactions with AT type and history of malignancy (Frontward time × AT type × Malignancy) and with AT type and vital status (Frontward time × AT type × Vital status). Within each modelling framework (LMM and GLMM), bold font indicates the best-fitting models, defined as those with the lowest AIC or BIC.

Supplementary Table 3. Estimated fixed-effects (and interactions) sizes, standard errors and levels of significance for selected linear mixed-effects models

LMM	Fixed effect (and interaction)	Effect size	Sd. error	*P*-adj
Frontward time	**Intercept**	23.754	2.483	$< 10^{-16}$
	**AT type (Variant AT)**	-9.037	2.128	$< 10^{-16}$
	ATM protein (Presence)	-3.35	1.933	0.528
	Malignancy (Yes)	-1.675	1.633	1
	Deceased (Yes)	-0.731	1.81	1
	**Time**	0.379	0.078	$< 10^{-16}$
Frontward time × Sex	**Intercept**	25.572	3.035	$< 10^{-16}$
	**AT type (Variant AT)**	-9.011	2.134	$< 10^{-16}$
	ATM protein (Presence)	-3.402	1.94	0.595
	Malignancy (Yes)	-1.944	1.656	1
	Deceased (Yes)	-0.543	1.82	1
	Time×Sex (Male)	0.275	0.124	0.224
	**Time×Sex (Female)**	0.435	0.093	$< 10^{-16}$
Frontward time × AT type	**Intercept**	24.567	2.864	$< 10^{-16}$
	**AT type (Variant AT)**	-10.436	3.212	0.014
	ATM protein (Presence)	-3.43	1.933	0.567
	Malignancy (Yes)	-1.698	1.63	1
	Deceased (Yes)	-0.618	1.814	1
	**Time×AT type (Classic AT)**	0.329	0.117	0.042
	**Time×AT type (Variant AT)**	0.412	0.096	$< 10^{-16}$
Frontward time × ATM protein	**Intercept**	27.902	3.91	$< 10^{-16}$
	**AT type (Variant AT)**	-9.15	2.123	$< 10^{-16}$
	ATM protein (Presence)	-8.147	3.952	0.301
	Malignancy (Yes)	-1.903	1.635	1
	Deceased (Yes)	-0.611	1.804	1
	Time × ATM protein (No)	0.136	0.191	1
	**Time × ATM protein (Presence)**	0.417	0.08	$< 10^{-16}$
Frontward time × Malignancy	**Intercept**	25.057	2.72	$< 10^{-16}$
	**AT type (Variant AT)**	-8.995	2.132	$< 10^{-16}$
	ATM protein (Presence)	-3.613	1.95	0.483
	Malignancy (Yes)	-4.822	3.196	0.952
	Deceased (Yes)	-0.973	1.824	1
	**Time×Malignancy (No)**	0.325	0.092	0.007
	**Time×Malignancy (Yes)**	0.505	0.135	$< 10^{-16}$
Frontward time × Vital status	**Intercept**	24.544	2.564	$< 10^{-16}$
	**AT type (Variant AT)**	-8.909	2.129	$< 10^{-16}$
	ATM protein (Presence)	-3.488	1.935	0.539
	Malignancy (Yes)	-1.82	1.636	1
	Deceased (Yes)	-4.348	3.425	1
	**Time × Vital status (Alive)**	0.33	0.087	$< 10^{-16}$
	**Time × Vital status (Deceased)**	0.537	0.148	0.007
Frontward time × AT type × Malignancy	**Intercept**	26.284	3.077	$< 10^{-16}$
	**AT type (Variant AT)**	-10.821	3.195	0.009
	ATM protein (Presence)	-3.826	1.913	0.45
	Malignancy (Yes)	-5.951	3.222	0.621
	Deceased (Yes)	-0.867	1.793	1
	Time×AT type (Classic AT) × Malignancy (No)	0.187	0.13	1
	**Time×AT type (Classic AT) × Malignancy (Yes)**	0.581	0.166	0.009
	**Time×AT type (Variant AT) × Malignancy (No)**	0.409	0.105	$< 10^{-16}$

	**Time × AT type (Variant AT) × Malignancy (Yes)**	0.486	0.147	0.018
Frontward time × AT type × Vital status	**Intercept**	26.894	3.14	$< 10^{-16}$
	**AT type (Variant AT)**	-12.315	3.357	$< 10^{-16}$
	ATM protein (Presence)	-3.721	1.937	0.54
	Malignancy (Yes)	-1.905	1.641	1
	Deceased (Yes)	-5.983	3.613	0.918
	Time × AT type (Classic AT) × Vital status (Alive)	0.172	0.147	1
	**Time × AT type (Classic AT) × Vital status (Deceased)**	0.506	0.156	0.018
	**Time × AT type (Variant AT) × Vital status (Alive)**	0.391	0.097	$< 10^{-16}$
	**Time × AT type (Variant AT) × Vital status (Deceased)**	0.646	0.205	0.027

Based on **Supplementary Table 2**, we considered models that included as fixed effects frontward time (i.e., disease duration, defined as the interval between age at diagnosis and age at assessment) or its second-order interactions with sex (Frontward time × Sex), AT type (Frontward time × AT type), ATM protein level (Frontward time × ATM protein), history of malignancy (Frontward time × Malignancy), and vital status (Frontward time × Vital status), as well as third-order interactions with AT type and history of malignancy (Frontward time × AT type × Malignancy) and with AT type and vital status (Frontward time × AT type × Vital Status). For main effects and interactions (significant results in bold fonts), we report the effect size, standard error, and adjusted *P*-value (*P*-adj).

Supplementary Table 4. Estimated fixed-effects (and interactions) log odds ratios, standard errors and levels of significance for selected generalised linear mixed-effects models

GLMM	Fixed effect (and interaction)	log(OR)	Std. error	*P*-adj
Frontward time	**Intercept**	0.809	0.265	0.012
	**AT type (Variant AT)**	-0.895	0.216	$< 10^{-16}$
	ATM protein (Presence)	-0.365	0.192	0.348
	Malignancy (Yes)	-0.156	0.164	1
	Deceased (Yes)	-0.115	0.185	1
	**Time**	0.039	0.008	$< 10^{-16}$
Frontward time × Sex	**Intercept**	0.969	0.322	0.021
	**AT type (Variant AT)**	-0.893	0.217	$< 10^{-16}$
	ATM protein (Presence)	-0.369	0.193	0.392
	Malignancy (Yes)	-0.183	0.168	1
	Deceased (Yes)	-0.096	0.187	1
	Time × Sex (Male)	0.03	0.013	0.168
	**Time × Sex (Female)**	0.043	0.01	$< 10^{-16}$
Frontward time × AT type	**Intercept**	0.979	0.305	0.007
	**AT type (Variant AT)**	-1.182	0.341	0.007
	ATM protein (Presence)	-0.381	0.193	0.336
	Malignancy (Yes)	-0.164	0.164	1
	Deceased (Yes)	-0.089	0.187	1
	Time × AT type (Classic AT)	0.028	0.013	0.168
	**Time × AT type (Variant AT)**	0.045	0.01	$< 10^{-16}$
Frontward time × ATM protein	**Intercept**	1.345	0.435	0.014
	**AT type (Variant AT)**	-0.908	0.218	$< 10^{-16}$
	ATM protein (Presence)	-0.963	0.433	0.182
	Malignancy (Yes)	-0.19	0.167	1
	Deceased (Yes)	-0.102	0.187	1
	Time × ATM protein (No)	0.008	0.021	1
	**Time × ATM protein (Presence)**	0.043	0.009	$< 10^{-16}$
Frontward time × Malignancy	**Intercept**	0.927	0.29	0.007
	**AT type (Variant AT)**	-0.892	0.217	$< 10^{-16}$
	ATM protein (Presence)	-0.388	0.194	0.322
	Malignancy (Yes)	-0.436	0.333	1
	Deceased (Yes)	-0.137	0.187	1
	**Time × Malignancy (No)**	0.034	0.01	$< 10^{-16}$
	**Time × Malignancy (Yes)**	0.05	0.014	$< 10^{-16}$
Frontward time × Vital status	**Intercept**	0.909	0.268	0.007
	**AT type (Variant AT)**	-0.88	0.215	$< 10^{-16}$
	ATM protein (Presence)	-0.385	0.191	0.308
	Malignancy (Yes)	-0.182	0.165	1
	Deceased (Yes)	-0.62	0.353	0.553
	**Time × Vital status (Alive)**	0.032	0.009	$< 10^{-16}$
	**Time × Vital status (Deceased)**	0.061	0.016	$< 10^{-16}$
Frontward time × AT type × Malignancy	**Intercept**	1.181	0.328	$< 10^{-16}$
	**AT type (Variant AT)**	-1.26	0.341	$< 10^{-16}$
	**ATM protein (Presence)**	-0.435	0.19	0.022
	Malignancy (Yes)	-0.628	0.34	0.065
	Deceased (Yes)	-0.112	0.184	0.543
	Time × AT type (Classic AT) × Malignancy (No)	0.013	0.014	0.368
	**Time × AT type (Classic AT) × Malignancy (Yes)**	0.055	0.017	0.001
	**Time × AT type (Variant AT) × Malignancy (No)**	0.046	0.011	$< 10^{-16}$

	**Time × AT type (Variant AT) × Malignancy (Yes)**	0.053	0.015	$< 10^{-16}$
Frontward time × AT type × Vital status	**Intercept**	1.354	0.329	$< 10^{-16}$
	**AT type (Variant AT)**	-1.498	0.354	$< 10^{-16}$
	ATM protein (Presence)	-0.438	0.194	0.216
	Malignancy (Yes)	-0.221	0.168	1
	Deceased (Yes)	-0.916	0.374	0.126
	Time × AT type (Classic AT) × Vital status (Alive)	0.005	0.015	1
	**Time × AT type (Classic AT) × Vital status (Deceased)**	0.054	0.016	0.009
	**Time × AT type (Variant AT) × Vital status (Alive)**	0.042	0.009	$< 10^{-16}$
	**Time × AT type (Variant AT) × Vital status (Deceased)**	0.087	0.02	$< 10^{-16}$

Based on **Supplementary Table 2**, we considered models that included as fixed effects frontward time or its second-order interactions with sex (Frontward time × Sex), AT type (Frontward time × AT type), ATM protein level (Frontward time × ATM protein), history of malignancy (Frontward time × Malignancy), and vital status of the study participant (Frontward time × Vital status), as well as third-order interactions with AT type and history of malignancy (Frontward time × AT type × Malignancy) and with AT type and vital status of the study participants (Frontward time × AT type × Vital status). For main effects and interactions (significant results in bold fonts), we report the log(OR), standard error, and adjusted *P*-value (*P*-adj).

Supplementary Table 5. Estimated Bayesian Information Criterion for each latent class mixed-effects model considered

		hlme				lcmm			
	**# Latent classes**	**# Parameters**	**BIC**	**% class1**	**% class2**	**# Parameters**	**BIC**	**% class1**	**% class2**
Frontward time	1	18	**1863.369**	100	-	23	**1739.527**	100	-
	2	21	1869.76	67.949	32.051	26	1746.163	24.359	75.641
Frontward time × Sex	1	19	**1865.873**	100	-	24	**1742.363**	100	-
	2	23	1876.536	62.821	37.179	28	1750.935	20.513	79.487
Frontward time × AT type	1	19	**1865.854**	100	-	24	**1741.035**	100	-
	2	23	1877.785	70.513	29.487	28	1734.498	91.026	8.974
Frontward time × ATM protein	1	19	**1863.762**	100	-	24	**1739.636**	100	-
	2	23	1875.494	84.615	15.385	28	1748.881	30.769	69.231
Frontward time × Malignancy	1	19	**1864.806**	100	-	24	**1740.69**	100	-
	2	23	1869.822	66.667	33.333	28	1745.781	69.231	30.769
Frontward time ×Vital Status	1	19	**1867.3**	100	-	24	**1743.647**	100	-
	2	23	1877.979	67.949	32.051	28	1755.762	75.641	24.359

We used the function hlme from the R package *lcmm* for the Box–Cox power transformation of the SARA score and the function lcmm for analyses using the original SARA score. To evaluate goodness of fit to the longitudinal data, we estimated the Bayesian Information Criterion (BIC) for models including frontward time or its second-order interactions with sex (Frontward time × Sex), AT type (Frontward time × AT type), ATM protein level (Frontward time × ATM protein), history of malignancy (Frontward time × Malignancy), and vital status of the study participants (Frontward time × Vital status), and for models specifying one or two latent classes. '# Parameters' indicates the total number of parameters estimated by hlme and lcmm. When two latent classes were specified, '% class 1' and '% class 2' report the proportion of patients assigned to each class. Within each modelling framework (hlme and lcmm), bold font indicates the best-fitting model with one or two latent classes.

Supplementary Table 6. Estimated Akaike Information Criterion and Bayesian Information Criterion for each mixed-effects model considered on a subset of probands with variant AT

		LMM				GLMM			
		Cross random effects		Nested random effects		Cross random effects		Nested random effects	
		**Frontward time**	**Quadratic function frontward time**	**Frontward time**	**Quadratic function frontward time**	**Frontward time**	**Quadratic function frontward time**	**Frontward time**	**Quadratic function frontward time**
Frontward time	AIC	949.5492	962.2478	**916.2596**	927.822	**952.307**	963.516	959.0445	960.5462
	BIC	991.1297	1007.027	**961.0386**	975.7995	**990.5556**	1005.097	1000.625	1005.325
Frontward time × Mutation	AIC	949.8298	963.2436	**916.8098**	933.99	**952.3972**	980.218	959.721	960.0243
	BIC	994.6087	1014.42	**964.7872**	988.3644	**993.8332**	1005.806	1004.5	1011.2
Frontward time × Mutation × Malignancy	AIC	957.7666	986.9498	**923.0199**	958.042	**956.2061**	963.516	963.5585	966.5564
	BIC	1008.943	1050.92	**977.3943**	1025.21	**1004.017**	1005.097	1014.734	1030.526
Frontward time × Mutation × Vital status	AIC	956.4723	986.2768	**922.1862**	954.8841	**947.3037**	958.244	954.7873	957.9472
	BIC	1007.648	1050.247	**976.5607**	1022.053	**995.1145**	1019.016	1005.963	1021.917

For a subset of patients with variant AT, we evaluated crossed and nested random-effects structures in linear mixed-effects models (LMMs) and generalized linear mixed-effects models (GLMMs). For each random-effects specification, we assessed whether frontward time or its quadratic term provided a better fit to the longitudinal data. For every model (defined by the mixed-effects framework (LMM or GLMM), random-effects structure (crossed or nested), and functional form of frontward time (linear or quadratic), we evaluated model fit using AIC and BIC. Rows in the table report the estimated AIC and BIC values for models including frontward time (or its quadratic term) and its second-order interactions with mutation status (Frontward time × Mutation), as well as third-order interactions with mutation and history of malignancy (Frontward time × Mutation × Malignancy) and vital status of the study participants (Frontward time × Mutation × Vital status). Within each modelling framework, bold font indicates the best-fitting model with the lowest AIC or BIC.

Supplementary Table 7. Estimated fixed-effects (and interactions) sizes, standard errors and levels of significance for selected linear mixed-effects models on a subset of probands with variant AT

LMM	Fixed effect (and interaction)	Effect size	Std. error	*P*-adj
Frontward time	**Intercept**	21.611	4.83	$< 10^{-16}$
	Mutation (Yes)	0.657	3.282	1
	Malignancy (Yes)	-2.675	3.247	1
	Deceased (Yes)	1.566	4.271	1
	**Time**	0.281	0.092	0.025
Frontward time × Mutation	**Intercept**	18.726	5.129	0.01
	Mutation (Yes)	8.052	5.360	1
	Malignancy (Yes)	-2.978	3.309	1
	Deceased (Yes)	2.08	4.341	1
	**Time × Mutation (No)**	0.397	0.111	0.006
	Time × Mutation (Yes)	0.075	0.15	1
Frontward time × Mutation × Malignancy	**Intercept**	18.603	5.289	0.008
	Mutation (Yes)	8.121	5.448	1
	Malignancy (Yes)	-1.569	5.345	1
	Deceased (Yes)	1.796	4.425	1
	**Time × Mutation (No) × Malignancy (No)**	0.43	0.137	0.032
	Time × Mutation (No) × Malignancy (Yes)	0.329	0.19	1
	Time × Mutation (Yes) × Malignancy (No)	0.063	0.16	1
	Time × Mutation (Yes) × Malignancy (Yes)	0.082	0.248	1
Frontward time × Mutation × Vital status	**Intercept**	18.819	5.249	0.01
	Mutation (Yes)	8.055	5.45	1
	Malignancy (Yes)	-2.889	3.407	1
	Deceased (Yes)	4.031	7.587	1
	**Time × Mutation (No) × Vital status (Alive)**	0.41	0.121	0.016
	Time × Mutation (No) × Vital status (Deceased)	0.27	0.298	1
	Time × Mutation (Yes) × Vital status (Alive)	0.064	0.158	1
	Time × Mutation (Yes) × Vital status (Deceased)	0.032	0.3	1

Based on **Supplementary Table 6**, for the subset of patients with variant AT, we considered models that included as fixed effects frontward time or its second-order interaction with mutation status (Frontward time × Mutation), as well as third-order interactions with mutation status and history of malignancy (Frontward time × Mutation × Malignancy) and with mutation status and vital status of the study participants (Frontward time × Mutation × Vital status). For main effects and interactions (significant results in bold fonts), we report the effect size, standard error, and adjusted *P*-value (*P*-adj).

Supplementary Table 8. Estimated fixed effects (and interactions) log odds ratios, standard errors and levels of significance for selected generalised linear mixed-effects models on a subset of probands with variant AT

GLMM	Fixed effect (and interaction)	log(OR)	Std. error	*P*-adj
Frontward time	**Intercept**	-0.01	0.482	1
	Mutation (Yes)	0.135	0.346	1
	Malignancy (Yes)	-0.419	0.345	1
	Deceased (Yes)	0.154	0.449	1
	**Time**	0.039	0.008	$< 10^{-16}$
Frontward time × Mutation	Intercept	-0.184	0.5	1
	Mutation (Yes)	0.619	0.495	1
	Malignancy (Yes)	-0.449	0.348	1
	Deceased (Yes)	0.152	0.452	1
	**Time × Mutation (No)**	0.047	0.010	$< 10^{-16}$
	Time × Mutation (Yes)	0.027	0.012	0.174
Frontward time × Mutation × Malignancy	Intercept	-0.211	0.506	1
	Mutation (Yes)	0.624	0.495	1
	Malignancy (Yes)	-0.398	0.453	1
	Deceased (Yes)	0.149	0.457	1
	**Time × Mutation (No) × Malignancy (No)**	0.05	0.012	$< 10^{-16}$
	**Time × Mutation (No) × Malignancy (Yes)**	0.044	0.014	0.008
	Time × Mutation (Yes) × Malignancy (No)	0.026	0.013	0.30
	Time × Mutation (Yes) × Malignancy (Yes)	0.028	0.021	1
Frontward time × Mutation × Vital status	Intercept	-0.451	0.443	1
	Mutation (Yes)	0.683	0.448	1
	Malignancy (Yes)	-0.548	0.297	0.512
	Deceased (Yes)	-2.022	0.789	0.08
	**Time × Mutation (No) × Vital status (Alive)**	0.041	0.010	< 10-16
	**Time × Mutation (No) × Vital status (Deceased)**	0.149	0.033	< 10-16
	Time × Mutation (Yes) × Vital status (Alive)	0.027	0.011	0.16
	**Time × Mutation (Yes) × Vital status (Deceased)**	0.098	0.029	0.008

Based on **Supplementary Table 6**, for a subset of individual patients with variant AT, we considered models that included as fixed effects frontward time or its second-order interaction with mutation status (Frontward time × Mutation), as well as its third-order interactions with mutation status and history of malignancy (Frontward time × Mutation × Malignancy) and with mutation status and vital status of the study participants (Frontward time × Mutation × Vital status). For main effects and interactions (significant results in bold fonts), we report the log(OR), standard error, and adjusted *P*-value (*P*-adj).
